# Density estimation and adaptive bandwidths: A primer for public health practitioners

**DOI:** 10.1186/1476-072X-9-39

**Published:** 2010-07-23

**Authors:** Heather A Carlos, Xun Shi, James Sargent, Susanne Tanski, Ethan M Berke

**Affiliations:** 1 Norris Cotton Cancer Center, Dartmouth-Hitchcock Medical Center, Lebanon, NH, USA; 2 Department of Geography, Dartmouth College, Hanover NH, USA; 3 Department of Community and Family Medicine, Dartmouth Medical School, Hanover NH, USA; 4 Prevention Research Center at Dartmouth, The Dartmouth Institute for Health Policy and Clinical Practice, Lebanon NH, USA

## Abstract

**Background:**

Geographic information systems have advanced the ability to both visualize and analyze point data. While point-based maps can be aggregated to differing areal units and examined at varying resolutions, two problems arise 1) the modifiable areal unit problem and 2) any corresponding data must be available both at the scale of analysis and in the same geographic units. Kernel density estimation (KDE) produces a smooth, continuous surface where each location in the study area is assigned a density value irrespective of arbitrary administrative boundaries. We review KDE, and introduce the technique of utilizing an adaptive bandwidth to address the underlying heterogeneous population distributions common in public health research.

**Results:**

The density of occurrences should not be interpreted without knowledge of the underlying population distribution. When the effect of the background population is successfully accounted for, differences in point patterns in similar population areas are more discernible; it is generally these variations that are of most interest. A static bandwidth KDE does not distinguish the spatial extents of interesting areas, nor does it expose patterns above and beyond those due to geographic variations in the density of the underlying population. An adaptive bandwidth method uses background population data to calculate a kernel of varying size for each individual case. This limits the influence of a single case to a small spatial extent where the population density is high as the bandwidth is small. If the primary concern is distance, a static bandwidth is preferable because it may be better to define the "neighborhood" or exposure risk based on distance. If the primary concern is differences in exposure across the population, a bandwidth adapting to the population is preferred.

**Conclusions:**

Kernel density estimation is a useful way to consider exposure at any point within a spatial frame, irrespective of administrative boundaries. Utilization of an adaptive bandwidth may be particularly useful in comparing two similarly populated areas when studying health disparities or other issues comparing populations in public health.

## Introduction

From John Snow's Victorian era map of cholera deaths [1] to interactive maps tracking the spread of H1N1 Influenza [2], spatial point patterns have a long and rich history in the public health arena. Disease registries now include geolocation data, which allow detection of clustering (a global tendency) and clusters (a local phenomenon). Public health practitioners focusing on disease prevention use spatial point pattern analysis to quantify social determinants of health (for example, distance to sites of physical activity [3] or to retail outlets [4], discrepancies in access to services by race or ethnicity, or variation in educational attainment).

Geographic information systems (GIS) have advanced the ability to both visualize and analyze these point data. Using GIS, point based maps can easily be aggregated to differing areal units and examined at varying resolutions. This however, creates problems in spatial analysis. In addition to introducing the modifiable areal unit problem (MAUP) [5], where altering the area or shape of an aggregate unit may alter the value within the polygon, any corresponding demographic data must also be available both at the scale of analysis and in the same geographic units. One way to address these issues is to employ kernel density estimation (KDE) techniques rather than geographic aggregation [6-8]. KDE is a non-parametric method of extrapolating point data over an area of interest without invoking MAUP or relying on fixed boundaries for aggregation. The density of points is calculated using a specified bandwidth (a circle of a given radius centered at the focal location). This produces a smooth, continuous surface where each location in the study area is assigned a density value, which can then be used as the independent or dependent variable in statistical models. KDE's strength is its ability to provide an estimate of density at any location in the spatial frame (e.g. a geocoded subject or another point of interest), irrespective of arbitrary administrative boundaries.

While various methods exist for calculating KDE surfaces, including some embedded in common GIS software, many public health practitioners and researchers use a static distance for bandwidth patterned after the case-control method [7,9]. A more in-depth discussion on the use of KDE in public health can be found in our prior work [10,11] and that of others [12]. When geographic distance (and count of cases) is the primary interest, a static bandwidth KDE appropriately represents the density of a particular attribute, for example understanding how relapse of alcoholism may be predicted in part by proximity to bars or pubs. However, for some health outcomes, a fixed geographic distance is not the appropriate bandwidth. Consider the hypothesis that alcohol outlets (retail alcohol sale locations) are more concentrated in low-income neighborhoods within a metro area. The problem with using a static bandwidth for each outlet is that we expect the greater density of alcohol outlets in urban areas where the population density is higher than in the suburbs. To the extent that outlet density in poor urban neighborhoods is just a reflection of a higher concentration of people living there, the correlation does not necessarily point to a health disparity.

There are a wide range of analytical methods available to examine spatial point patterns [12] and other researchers have considered the effect of inhomogeneous background populations. Here, inhomogeneous background populations refers not to the population's demographics, rather to the distribution of the source of the event points. For our alcohol outlet example, the background population is the population of the study area, whereas in an analysis of disease cases, only the population at risk is used. Notably, the spatial filtering technique [13,14] uses both fixed and adaptive filters/bandwidths to test or map the relationship between a count of cases and a background population while the cluster evaluation permutation procedure [15,16] uses an adaptive bandwidth but focuses only on the case count. Alternatively, adding population density as a covariate in a statistical model could address this issue, but a more elegant solution incorporates population density into the outcome variable using a bandwidth that represents the underlying population, rather than a fixed geographical area. An adaptive bandwidth, discussed below, may be preferable when studying issues of population and variations in exposure.

In this paper, we present a number of approaches to density estimation and propose using a KDE method to address uneven population distribution by using an adaptive bandwidth specified by the underlying population. This technique is useful when it is it is important to understand if a density value is just a reflection of the local population or if it may stem from other causes. This methodology was motivated by an analysis of the distribution of alcohol outlets. An illustrative application in that arena is used to compare density methods, but it is also applicable to the analysis of the density of disease, crime, healthcare clinics and other fields where the background population is heterogeneous.

## Background

We focus on a suite of density estimation tools: point density, static bandwidth kernel density estimation and adaptive bandwidth kernel density estimation. Density calculations operate on either *cases *or *sites*. Cases are event points (e.g. addresses of alcohol outlets or disease cases) whereas sites represent all locations (pixels or each point on a grid) in a study area. Density calculations performed on sites (site-side method) evaluate the density for every location in the study area, whereas the case-side method only looks at case locations and their defined surrounding locations. In order to highlight the differences in the density estimation tools presented here, we limit our discussion to case-side methods of density estimation. Case-side methods are more computationally efficient and in many situations better represent the nature of the application problem. More information about the differences between case-side and site-side methods can be found in Shi [11].

### Point Density

**Figure 1:**
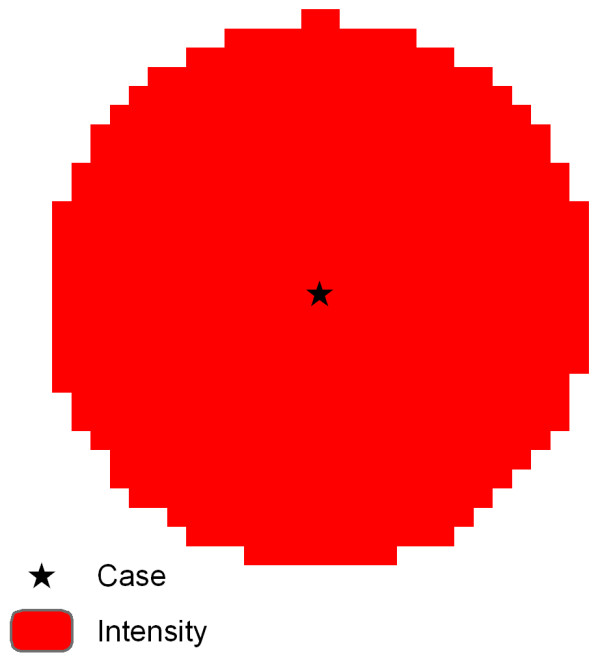
**Point density**. Equal values at all locations within the neighborhood (the circle) around the case (star in center).

Most generically, a point density function (also called intensity function) defines the number of cases (alcohol outlets, disease cases) per unit area at each location throughout an area of interest. To calculate this density surface, for each case, a "neighborhood" is delineated, usually by defining a search radius (or bandwidth); the number of cases that fall within the neighborhood are divided by the area of the neighborhood; this value is assigned to the neighborhood (Figure 1). The intensity function is expressed as [17](1)

where λ(x,y) is the intensity (or point density) at location(x,y), n is the number of events and |A| is the area of the neighborhood. When neighborhoods overlap, the results are summed to indicate a higher density of cases. The units of λ(x,y) are cases per unit area.

When points are evenly distributed in space, increasing the bandwidth does not have a large impact on the intensity since, as larger neighborhoods are defined, n will likely increase, but so will |A|. However, increasing the bandwidth does provide a greater smoothing effect (or a more generalized surface), which risks removing meaningful spikes (peaks or valleys) or edges (extent of the influence of a case) from the original data distribution.

Although the point density function is relatively simple and straightforward, it does not convey any information about the spatial configuration of features of interest within the bandwidth. Consider two locations (sites) and one case. Computationally, a site coincident with a case returns the same λ as a site one bandwidth away from the case. While this approach is appropriate for studies which are interested in the number of events per unit area at a specified location (e.g. crime events, residential or population density), in other disciplines there is an expected attenuation with distance (e.g. environmental pollutants which dissipate as they travel from the source). In order to compensate for distance, a density function can incorporate a decay function to assign smaller values to locations which are still in the neighborhood, but more distant from a case. This is the approach employed by kernel density estimation.

### Static Bandwidth Kernel Density Estimation

**Figure 2:**
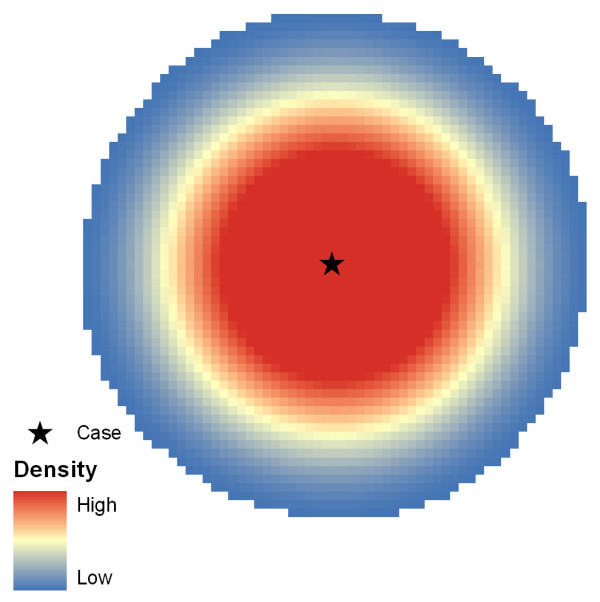
**Kernel Density Estimation**. The decay function is illustrated with the highest values located under the case giving way to lower values.

**Figure 3:**
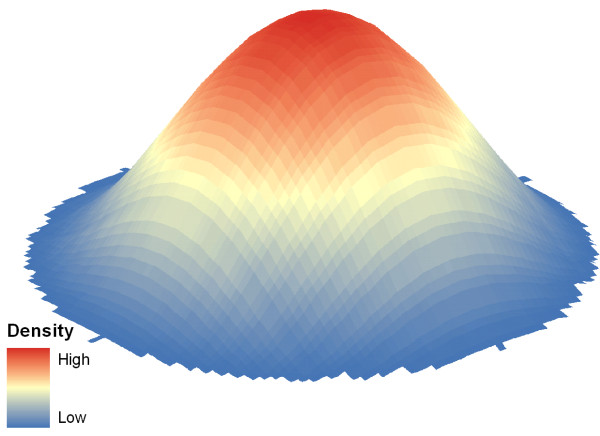
**3 D rendering of KDE**.

Kernel density estimation fits a curved surface over each case such that the surface is highest above the case and zero at a specified distance (the bandwidth) from the case (Figures 2 and 3). In mathematical terms, it is expressed as [6](2)

where f(x,y) is the density value at location (x,y), n is the number of cases, h is the bandwidth, d_i _is the geographical distance between case i and location (x, y) and K is a density function (generally a radially symmetric unimodal probability density function) which integrates to one. The units of f(x,y) are cases per unit area.

Static bandwidth kernel density estimation is a technique that is appropriate when geographic distance (and case count) is the primary concern. Since it applies the same geographic extent to each case, static bandwidth KDE does not distinguish the spatial extents of interesting areas [11], nor does it expose patterns above and beyond those due to geographic variations in the density of the underlying population [17].

### Problems caused by heterogeneous backgrounds

Because health outcomes involve people, their spatial distribution will often reflect the spatial distribution of the underlying human population. Counts of disease are almost always higher in urban areas than rural areas simply due to the size of the potential exposed population. Likewise, counts of things people use are greater in higher population areas: there are more parks, physical activity sites, and retail outlets in places where more people live. As a result, the density of occurrences should not be interpreted without knowledge of the underlying population distribution [7]. When the effect of the background population is successfully accounted for, differences in point patterns in similar population areas are more discernible; it is generally these variations that are of most interest [18].

**Figure 4:**
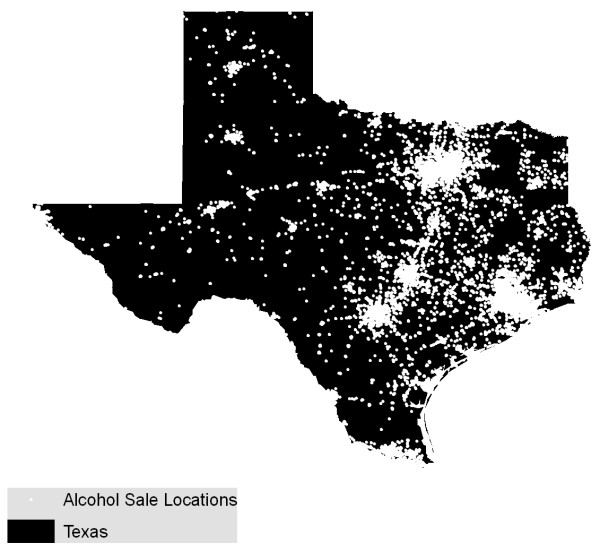
**Map of alcohol outlets**.

**Figure 5:**
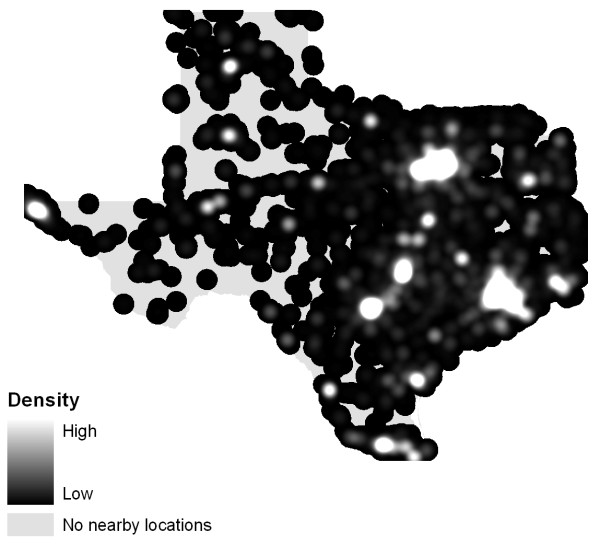
**Kernel Density Estimation of alcohol outlets**.

**Figure 6:**
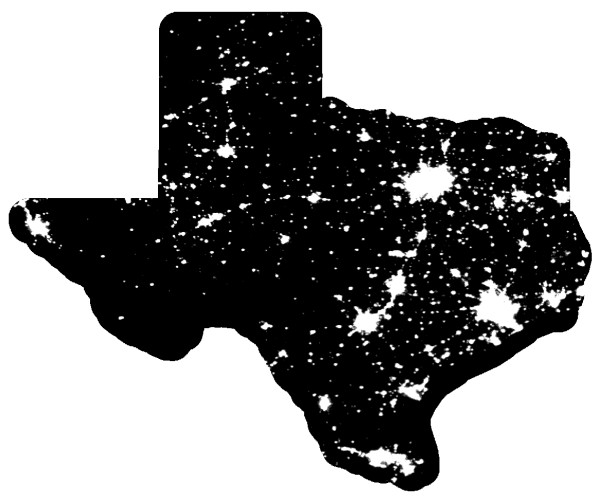
**Texas at night, as seen from space**[23].

**Figure 7:**
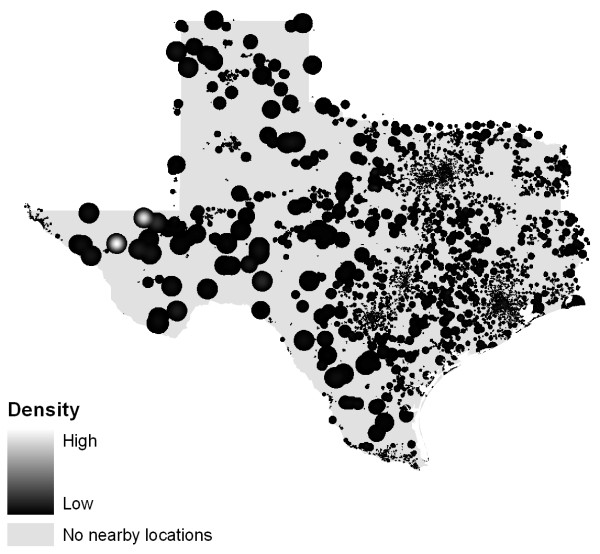
**Adaptive Bandwidth Kernel Density Estimation of alcohol outlets**.

A Texas case study illustrates the problem posed by heterogeneous population backgrounds. Figure 4 displays the point data for alcohol outlets while Figure 5 shows the static bandwidth KDE surface for these data. As we would expect, both maps are similar to an image of Texas at night (Figure 6) since they replicate the population distribution. In contrast, Figure 7 shows a density map with the underlying population addressed. An adaptive bandwidth KDE method was used to create this map and it is described below.

## Methods - Adaptive Bandwidth

### Data Sources

Before delving into the adaptive bandwidth methodology, we discuss a data source for the background population. Most GIS based population data are in a polygon format with a population count (or estimate) assigned to each polygon. Depending on the study area and data source, each polygon may be as large as a country, or as small as a city block. These polygons are often irregular shapes and sizes and lack data about how people are geographically dispersed within the polygon. In addition, administrative boundaries may not be consistent with the travel patterns or service utilization of those that live in them. An alternative to polygon based population data is the LandScan™ Global Population Database, which was developed using multiple techniques to disaggregate census counts within an administrative boundary. This worldwide population data product is available on a 30" × 30" latitude/longitude grid (a pixel located in the central United States is approximately 0.65 km^2^). The advantage of the grid format is that it regularizes the areal unit for population values, unlike administrative boundaries, which vary in size. This makes counts at different locations more spatially comparable and facilitates spatial analysis operations. However, it disconnects the population counts from the related demographic data, which is included in many censuses. Additionally, in urban areas, census blocks may be smaller than the LandScan™ grid and as a result, the larger grid units aggregate the original population counts. A description of the LandScan™ data and the methodology used to create it are described in detail elsewhere [19,20].

Geocoded data for the alcohol outlets were obtained from the NAICS (North American Industry Classification System) Association http://www.naics.com. Details on this data and related processing can be found elsewhere [4].

### Adaptive Bandwidth Kernel Density Estimation

**Figure 8:**
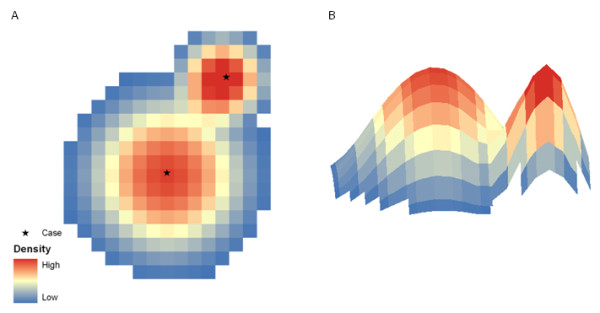
**Two overlapping cases and the related density surface**. In A, the case in the upper right has a smaller spatial impact, as the expected population was reached earlier than the case in the lower left. B is a rotated 3 dimensional image of the surface shown in A.

Whereas the static bandwidth kernel density estimation model employs a bandwidth based on a geographic distance, the adaptive bandwidth method uses background population drawn from LandScan™ data to calculate a kernel of varying size for each individual case (which, using the examples above could be an alcohol outlet). This limits the influence of a single case to a small spatial extent where the population density is high as the bandwidth is small [10]. Conversely, in rural areas where population is lower, the kernel is geographically larger and the influence of a single case is greater (Figure 8).

The adaptive method is calculated as follows [10]:(3)

Note that there are several differences between equations 2 and 3. Most notably, in the adaptive method, the bandwidth is represented by P(u,v) which is a function centered on the case located at (u,v) and based on the local population. Additionally, the denominator nh^2 ^is dropped since the output value is not normalized by the geographic area of the kernel (h^2^). The adaptive bandwidth method bases the influence of a case on the underlying population support, not the area support. There are various choices for the function K; most will not significantly affect the outcome. This study uses a simple form:(4)

Often the influence of one case will overlap that of another. In this situation, the bandwidth and kernel density estimation calculations are performed separately for each case and then the results are summed (Figure 8, Equation 3).

### Constraints on the Adaptive Bandwidth

The expected population parameter determines the extent of the adaptive bandwidth. The expected population normalizes the influence of each case to a certain number of people and thus the bandwidth stops expanding when the expected population is reached. In less populous areas however, the bandwidth could expand beyond the distance where a case may influence health; one can therefore set a limit to the maximum distance of the bandwidth. The maximum distance parameter restricts the bandwidth from expanding further, even if the expected population has not been reached. This may be critical when considering health behaviors influenced by exposures that are beyond a reasonable distances from an individual.

Bandwidth is determined for each individual case by summing the underlying population, starting with the pixel directly under the case and then expanding outward until the expected population is reached. Given reasonable values for population and maximum distance, expected population exerts the most control. In urban areas with high population densities, the expected population limit will often be reached before the maximum distance, thus, by adjusting the expected population, the radius of the influence of the case will diminish. The same is true in rural areas, except that in areas of very low population density the maximum distance may be called into play to limit bandwidth.

## An Application of Adaptive Bandwidth

### Static and Adaptive Bandwidth KDE for Alcohol Outlets

**Figure 9:**
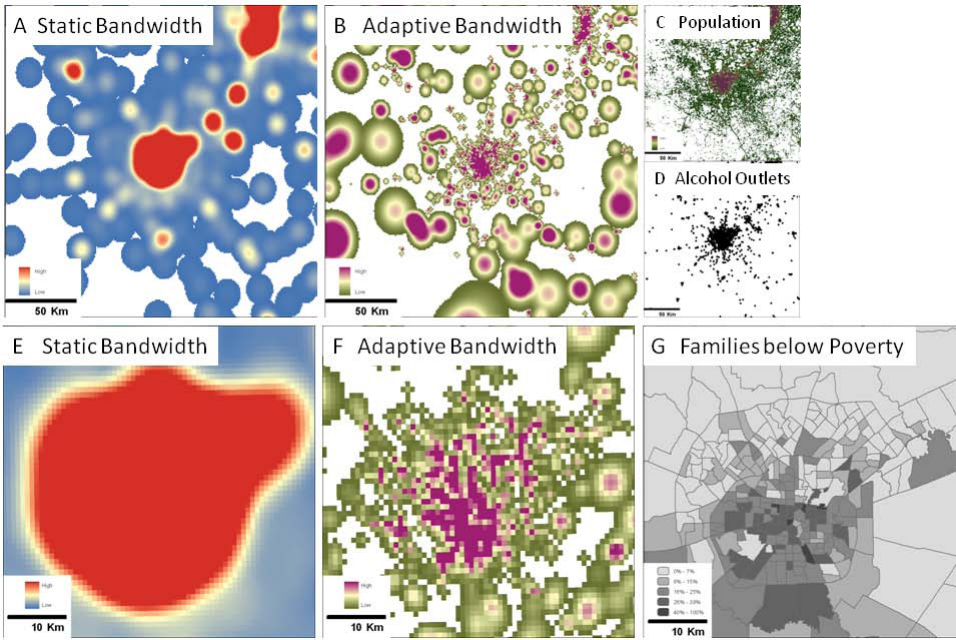
**Cartographic comparison of density estimation of alcohol outlets near San Antonio, Texas**. A-D show San Antonio in the center and Austin in the upper right with more rural areas to the south and west, E-G are zoomed in to show just San Antonio. A and E illustrate KDE using a static bandwidth of ~10 km. B and F illustrate KDE using an adaptive bandwidth with an expected population of 1,000 people and a maximum distance of ~25 km. C shows a LandScan™ dataset where each pixel represents a population count. D shows point data representing alcohol outlets. G is a map of census tracts showing the percentage of families below the poverty level.

The difference between static and adaptive bandwidth KDE methods is best illustrated through visualization. Figure 9 portrays the results from each method applied to alcohol outlets (Figure 9D) in the area surrounding San Antonio, Texas. As mentioned above, the static bandwidth KDE surfaces (Figures 9A and 9E) excel at identifying areas where there are many point sources, but they do not provide a basis for discerning where the point sources are higher or lower than would be expected given the underlying population (Figure 9C). The adaptive bandwidth KDE (Figure 9B and 9F) addresses these issues through utilization of a population-based bandwidth, allowing for improved detection of neighborhood-level differences in exposure, even in areas that have similarly high raw counts of alcohol outlets. This is illustrated in the close-up of San Antonio (Figures 9E and 9F) where the adaptive bandwidth KDE (Figure 9F) shows fine-grained variability in the urban center whereas the static bandwidth KDE (Figure 9E) shows little differentiation in alcohol outlet density. This level of analysis is important in associating density of exposure with markers of health disparities, such as poverty, as seen in Figure 9G.

In deciding which method to choose, one needs to consider the research hypothesis. Using alcohol outlets as an example, if the primary concern is distance, a static bandwidth is preferable because it may be better to define the "neighborhood" or exposure risk of each store based on distance. If the primary concern is the "share" of the "service" per person, or differences in exposure across the population, a bandwidth adapting to the population is preferred.

More theoretically, the static bandwidth has a fixed spatial certainty, but varying statistical stability across the area, and thus is more suitable for an application primarily concerning distance. The adaptive bandwidth has a fixed statistical stability but varying spatial certainty. As a result, in a high population density area, it has both high statistical stability (specified by the user) and high spatial certainty (e.g., can better reveal the size of a hot spot), but in a low population density area, its high statistical stability comes with a cost of low spatial certainty.

### Limitations

There are limitations to all methods of spatial analysis, including density estimation, which induces interpolation autocorrelation which may result in over smoothing [21] (this can be controlled by using a global Monte Carlo simulation). Perhaps the greatest limitation is the relatively arbitrary selection of bandwidth limits with both static and adaptive methods. Too large or small a bandwidth poses the risk of over or undersmoothing the original data, respectively. As subsequent analyses are based on this estimated density information, as opposed to original points, a change in bandwidth may have a significant impact on statistical relationships between dependent and independent variables. Methods to estimate appropriate bandwidths are described in more detail elsewhere [6,17,22]. We recommend, even when applying mathematical models to estimate bandwidth, that one test multiple parameters for bandwidth in a sensitivity analysis. When employing a population-based adaptive bandwidth, applying a distance limit similar to the example above may be useful when considering the influence of exposure on behavior. In the example of alcohol outlets used above, we placed a 25 km limit on the density calculation if the expected population threshold of 1000 people was not reached. We determined this maximum distance by testing a number of distances as well as choosing a limit based on behavior theory regarding alcohol acquisition and consumption. For a process other than alcohol exposure, a different distance limit may make more sense.

Even though techniques such as static or adaptive bandwidth KDE do not rely on aggregated data or administrative boundaries, issues of data visualization remain. One should use caution when viewing density data at a small scale. This is particularly true when viewing U.S. national maps where variations in density in high population but geographically smaller, northeastern areas are difficult to visualize, leading to visual bias towards the lower population areas of the west. Indeed, display of a density map may not be appropriate at all unless the proper scale is chosen. Quantitative data from the KDE may be better reported in a tabular presentation. Finally, the analyses in this study were conducted using a program created by one of the authors, as opposed to commercially available software. With recognition of multiple methods of KDE and its use on the rise, we expect that these approaches will appear in common GIS and spatial statistics software in the near future.

## Conclusion

Researchers in the health sciences should be aware that multiple approaches to density estimation exist. Kernel density estimation is a useful way to consider exposure at any location within a spatial frame, irrespective of administrative boundaries. The ability for the researcher to analyze data easily at multiple scales reduces the risk of misinterpretation of results due to the MAUP. Utilization of an adaptive bandwidth may be particularly useful in comparing two similarly populated areas when studying health disparities.

## Competing interests

The authors declare that they have no competing interests.

## Authors' contributions

HC conceived of the study, performed the analysis, and drafted the manuscript. XS designed and implemented the population based adaptive bandwidth method and assisted with manuscript preparation. JS assisted with data interpretation and manuscript preparation, ST assisted with data interpretation and manuscript preparation. EB conceived of the study, supervised analyses, and drafted the manuscript. All authors read and approved the final manuscript.
